# Gastrodin inhibits the formation of ataxin-3 aggregates by regulating the level of ERK1/2/P38 proteins

**DOI:** 10.1186/s13023-025-04089-1

**Published:** 2026-02-19

**Authors:** Zijian Wang, Xunhao Xiao, Min Wang, Ruitong Cheng, Chan Wang, Yingxun Liu, Fengqin He, Xiaodong Xie

**Affiliations:** 1 Key Laboratory of Natural Product Development and Anticancer Innovative Drug Research in Qinling, School of Biological and Environmental Engineering, Xi’an University, Xi’an, Shaanxi 710065 China; 2 College of Animal Science and Technology, Yangtze University, Jingzhou, 434025 China; 3 Institute of Genetics, School of Basic Medical Sciences, Lanzhou University, Lanzhou, 730000 China

**Keywords:** Gastrodin, SCA3, Ataxin-3 aggregation, ERK/p38 signaling, Neuroprotection

## Abstract

**Background:**

Spinocerebellar ataxia type 3 (SCA3/Machado-Joseph disease), an incurable autosomal dominant neurodegenerative disorder, is caused by cytotoxic aggregation of polyglutamine-expanded ataxin-3 protein. Novel therapeutic strategies targeting its pathogenesis are urgently needed.

**Purpose:**

Given gastrodin’s established antioxidative and neuroprotective properties, this study investigated its therapeutic potential against SCA3 pathogenesis.

**Methods:**

Three distinct cell models including parental HEK293T, ataxin-3-15Q (physiologic), and ataxin-3-77Q (pathogenic) were employed to assess gastrodin cytotoxicity, quantify insoluble aggregate formation and measure soluble ataxin-3 levels. Mechanistic studies included antioxidant capacity assays, human phosphokinase array profiling (37 kinases) and western blot validation of MAPK pathway components.

**Results:**

Gastrodin treatment showed no cytotoxicity, significantly suppressed ataxin-3-77Q aggregate accumulation (*p* < 0.01), increased soluble ataxin-3 levels, enhanced cellular antioxidant capacity and selectively downregulated ERK1/2 and p38 proteins in MAPK pathways.

**Conclusion:**

We provide first evidence that gastrodin mitigates polyQ-mediated proteotoxicity by reducing ataxin-3 aggregation through suppression of the ERK1/2-p38 signaling axis in cellular models, revealing a novel mechanistic basis for SCA3 therapeutic development.

**Supplementary Information:**

The online version contains supplementary material available at 10.1186/s13023-025-04089-1.

## Highlights

Gastrodin’s safety profile was demonstrated in SCA3 cellular models at concentrations up to 100 µM without causing cytotoxicity.

Gastrodin significantly reduced the formation of polyQ-expanded ataxin-3 aggregates.

There was a dose-dependent increase in soluble ataxin-3 levels by gastrodin.

Gastrodin was found to attenuate SCA3 proteotoxicity in cellular models by simultaneously decreasing the total protein levels of ERK1/2 and p38.

**Supplementary Information:**

The online version contains supplementary material available at 10.1186/s13023-025-04089-1.

## Introduction

Spinocerebellar ataxia type 3 (SCA3/Machado-Joseph disease), the most prevalent autosomal-dominant inherited ataxia globally [1, 2], arises from abnormal polyglutamine (polyQ) expansion in ataxin-3. This mutation triggers cytotoxic protein aggregation-a hallmark of polyQ disorders - where misfolded ataxin-3 forms insoluble inclusions that sequester vital cellular components and drive neurodegeneration [3, 4]. Critically, no disease-modifying therapies currently exist to halt SCA3 progression [5].

### Pathogenesis and Current Therapeutic Challenges

Pathogenic synergy between proteotoxicity and oxidative stress underpins SCA3 pathogenesis. PolyQ-expanded ataxin-3 directly impairs proteostasis by overwhelming autophagy and ubiquitin-proteasome systems [6]. Concurrently, it induces mitochondrial dysfunction and redox imbalance, evidenced by elevated oxidative stress markers and diminished antioxidant defenses in patients and models [7]. This dual insult creates a vicious cycle: oxidative stress accelerates aggregate formation, while aggregates further amplify oxidative damage. Existing therapeutic strategies targeting aggregation (e.g., autophagy enhancers, proteasome activators) or oxidative stress show preclinical promise but face translational barriers including safety concerns and poor blood-brain barrier (BBB) penetration [8]. Repurposing phytochemicals with established neuroprotective profiles offers a pragmatic alternative [9].

Gastrodin, the primary bioactive compound from Gastrodia elata Blume, emerges as a compelling candidate due to its multimodal neuroprotection, including demonstrated antioxidant, anti-aggregation, and anti-apoptotic effects in Alzheimer’s and Parkinson’s models [10–12]; its favorable pharmacokinetics, including proven blood-brain barrier permeability with minimal side effects [13]; and its unmet potential, never before evaluated in polyQ disorders. Here, we bridge this gap by investigating gastrodin’s efficacy against SCA3 proteotoxicity. Using HEK293T-based models expressing physiologic (15Q) or pathogenic (77Q) ataxin-3, we assessed its effects on cell viability, aggregate burden, and soluble ataxin-3 levels, mapped kinase signaling alterations via phosphoproteomic screening, mechanistically dissected its regulation of the ERK1/2-p38 axis - a convergence point for oxidative stress and proteostasis disruption.

## Results

### Gastrodin at concentrations ranging from 5 µM to 100 µM is safe in HEK293T cells overexpressing ataxin-3

**Fig. 1:**
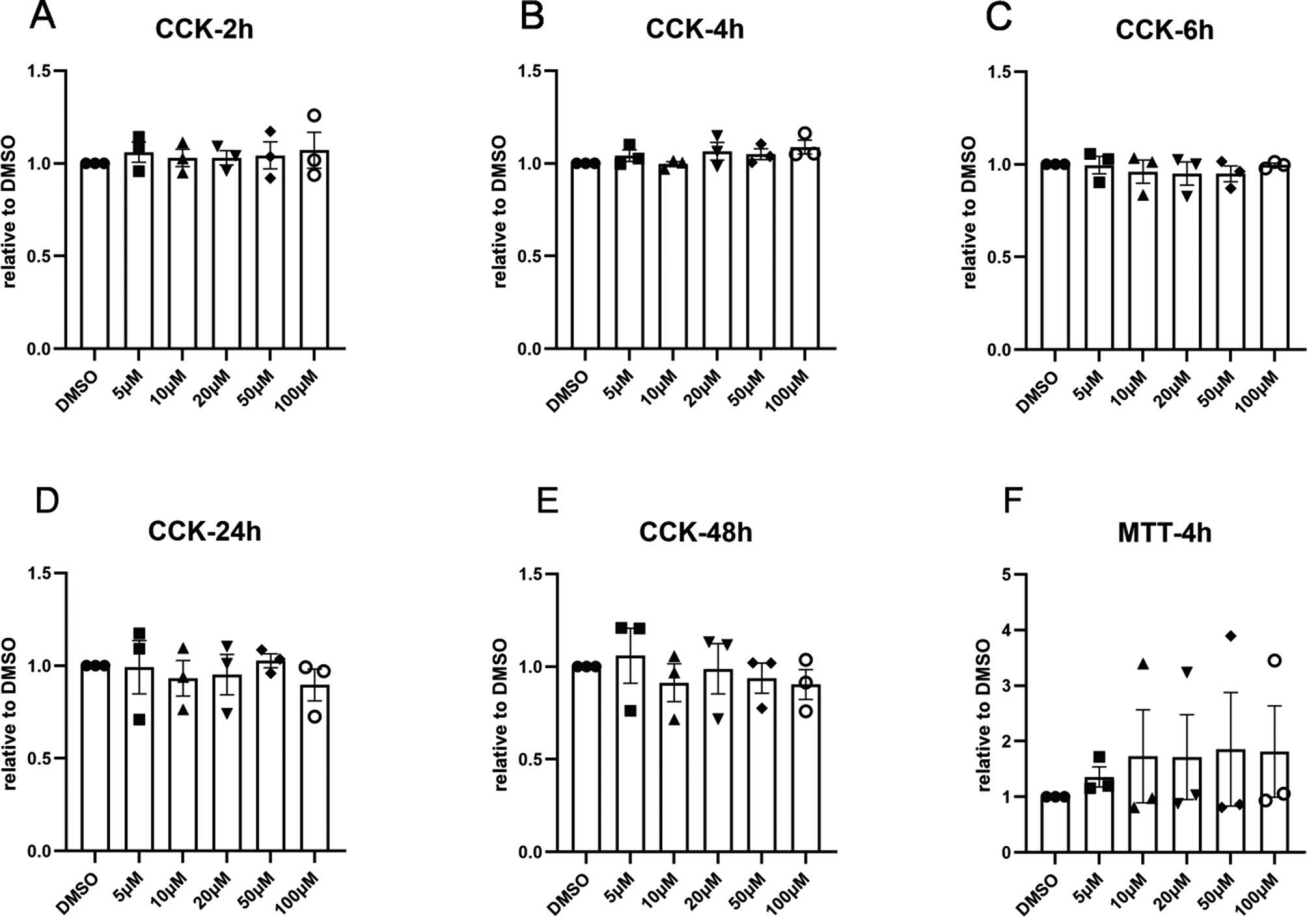
Gastrodin does not affect cell viability in an SCA3 cell model. HEK293T cells overexpressing ataxin-3-77CAG were treated with different concentrations of gastrodin for 2, 4, 6, 24, or 48 h as measured by CCK-8 (**A**-**E**) and for 4 h as measured by MTT (**F**). The x-axis displays the treatment groups, while the y-axis shows the OD values normalized to the DMSO-treated group. The data were collected in triplicate for three independent experiments (*n* = 3). Error bars indicate the standard error of the mean. The effects of each gastrodin concentration on DMSO-treated cells were analyzed using one-way ANOVA

To investigate the effect of gastrodin on cellular toxicity in SCA3 cell models, two methods, the CCK-8 method and the MTT assay, were used to assess cell proliferation and cell toxicity. CCK-8 assays revealed that gastrodin at concentrations of 5-100 µM did not have cytotoxic effects on cells after 2, 4, 6, 24, or 48 h (Fig. 1A-E). The MTT results were consistent with the CCK-8 assay results (Fig. 1F). These findings indicate that gastrodin is considered safe for use in these SCA3 cell models, with concentrations ranging from 5 to 100 µM.

### Gastrodin reduces polyQ-expanded ataxin-3 aggregates

**Fig. 2:**
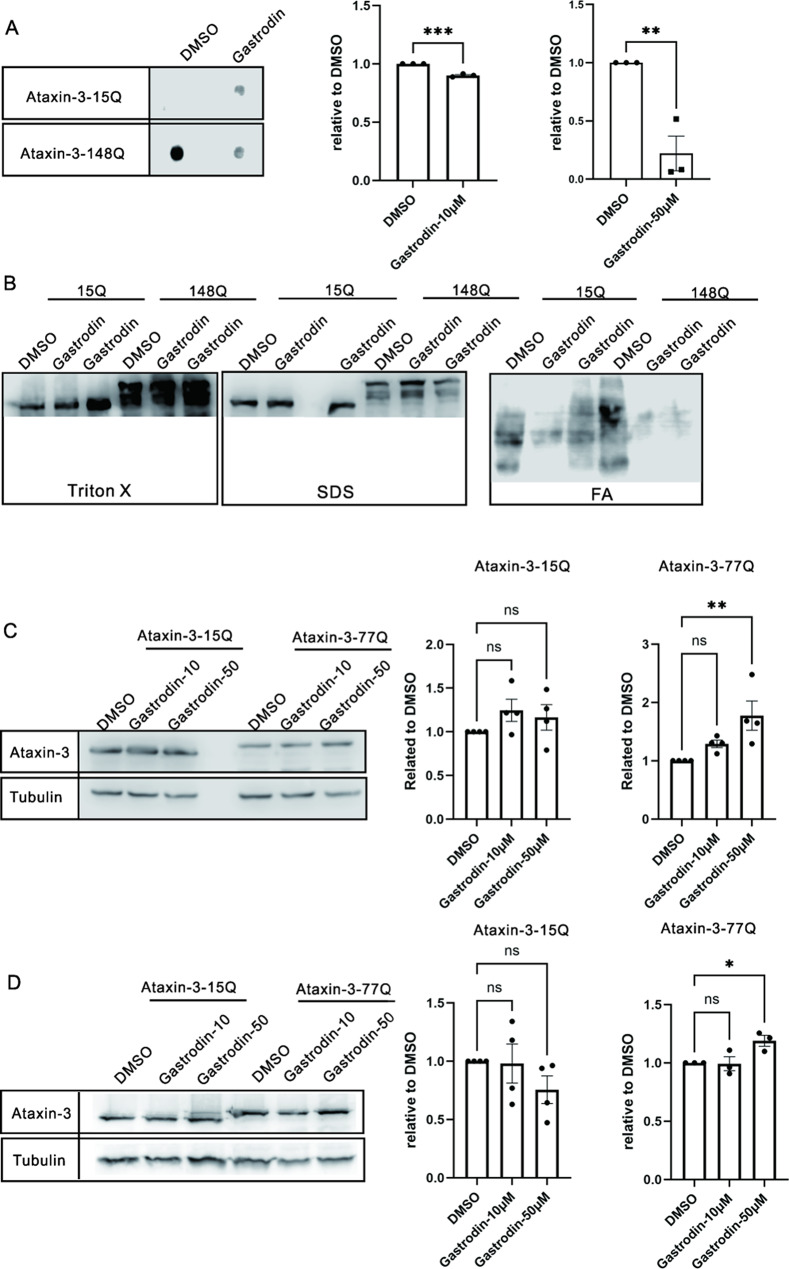
**A**. Treatment with 50 µM of gastrodin decreased the total amount of aggregated ataxin-3, as shown by filter trap assay results. Representative dot images of filter traps are displayed. Bar graphs indicate values relative to the DMSO-treated group (mean ± SEM, *N* = 3). **B**. Cell fractionation was performed according to Koch et al. [16]. Gastrodin treatment tended to decrease the amount of formic acid (FA)-soluble aggregates (large inclusions) and increase the amount of Triton X-100 (soluble protein) and SDS soluble aggregates (small inclusions) of expanded ataxin-3. **C**-**D**. Gastrodin at concentrations of 10 µM or 50 µM was tested for its effect on soluble ataxin-3 protein levels after 4 h (**C**) or 24 h of treatment (**D**). Western blot images are provided. Bar graphs illustrate the impact of gastrodin on normal ataxin-3 (15Q) and expanded ataxin-3 (77Q) (mean ± SEM, *N* = 4). The x-axis in panel **A** and **C**-**D** shows different treatment groups. The y-axis in panel A represents ataxin-3 signal (1H9) normalized to the DMSO-treated group. The y-axis in panel **C**-**D** represents ataxin-3 signal (1H9) relative to the loading control tubulin normalized to the DMSO-treated group. One-way ANOVA and multiple comparisons were conducted

To explore the effect of gastrodin on attenuating the aggregates formed by expanded ataxin-3 in a cell model of SCA3, a filter trap assay was employed, and a cell fractionation analysis was performed. Based on the cytotoxicity results and reports in the literature, we selected a gastrodin concentration of 10 µM or 50 µM, which has a protective effect on other neurodegenerative diseases [14, 15]. Filter trap assessment revealed that, compared with DMSO treatment, gastrodin treatment significantly decreased the amount of aggregates formed by expanded ataxin-3 (Fig. 2A).

To analyze the fractions of aggregates decreased by gastrodin, we fractionated the aggregated ataxin-3 using a fractionation assay. Consistently, treatment with gastrodin decreased the amount of insoluble expanded ataxin-3 aggregates observed in formic acid (FA)-treated cells, which is an SDS-insoluble protein that is harmful to cells. Moreover, after gastrodin treatment, the levels of the Triton X-100-soluble (soluble protein) and SDS-soluble ataxin-3 proteins (small aggregates) increased, indicating that promotes the dissolution or disassembly of large aggregates into more soluble forms (Fig. 2B).

Further analyses were conducted to investigate the effect of gastrodin on the total level of soluble ataxin-3 protein. Western blot analysis revealed that 10 or 50 µM gastrodin treatment for 4–24 h significantly increased soluble expanded ataxin-3 levels, while exerting minimal effects on normal ataxin-3 (Fig. 2C, D). These findings suggest that gastrodin effectively prevents aggregate formation in expanded ataxin-3-expressing HEK293T cells.

### Gastrodin exerts a protective role via the ERK1/2 and p38 signaling pathways

**Fig. 3:**
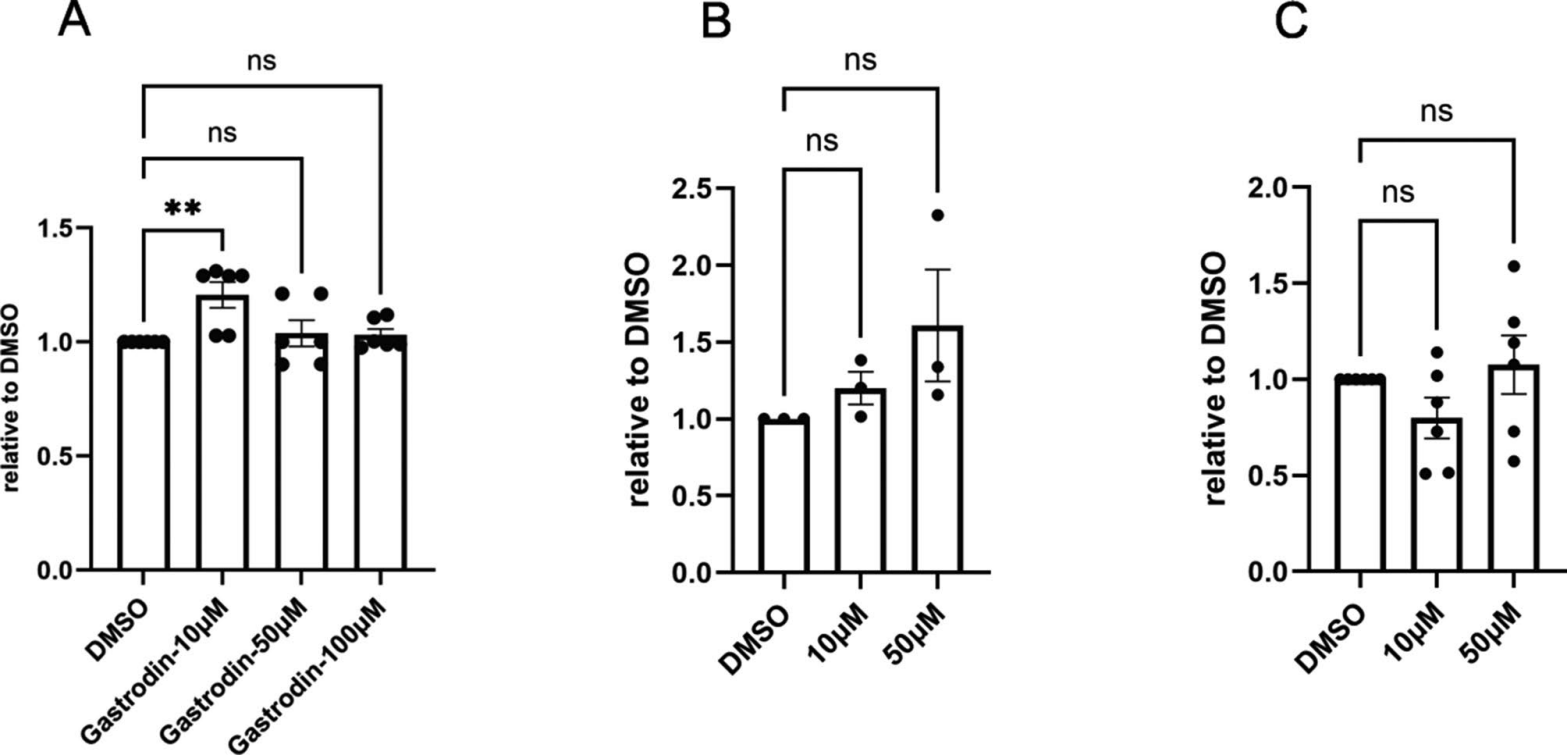
Effects of different concentrations of gastrodin on the antioxidant activity in (**A**) HEK293T cells, (**B**) HEK293T cells overexpressing ataxin-3-15Q, and (**C**) HEK293T cells overexpressing ataxin-3-77Q. The x-axis displays the various treatment groups, while the y-axis indicates the total antioxidative activity of the samples compared to the DMSO-treated group using a standard curve with Trolox for normalization. Data are based on more than three independent experiments (*n* ≥ 3) with technical duplicates, analyzed using one-way ANOVA and multiple comparisons. The significance level was set at *P* < 0.01**

To investigate the mechanism by which gastrodin inhibits protein aggregation, we first used the total antioxidant capacity assay to assess the antioxidative ability of gastrodin in three cell models. Consistent with previous reports, compared with DMSO treatment, 10 µM gastrodin treatment increased the antioxidative activity in HEK293T cells, 10 µM and 50 µM gastrodin treatment showed an increasing trend of the antioxidative activity in ataxin-3-15Q-expressing HEK293T cells (Fig. 3A, B). However, gastrodin did not exhibit antioxidative activity in ataxin-3-77Q-expressing HEK293T cells (Fig. 3C). These data demonstrated that while gastrodin possesses antioxidant activity, it may also confer protection through additional signaling pathways in SCA3.

**Fig. 4:**
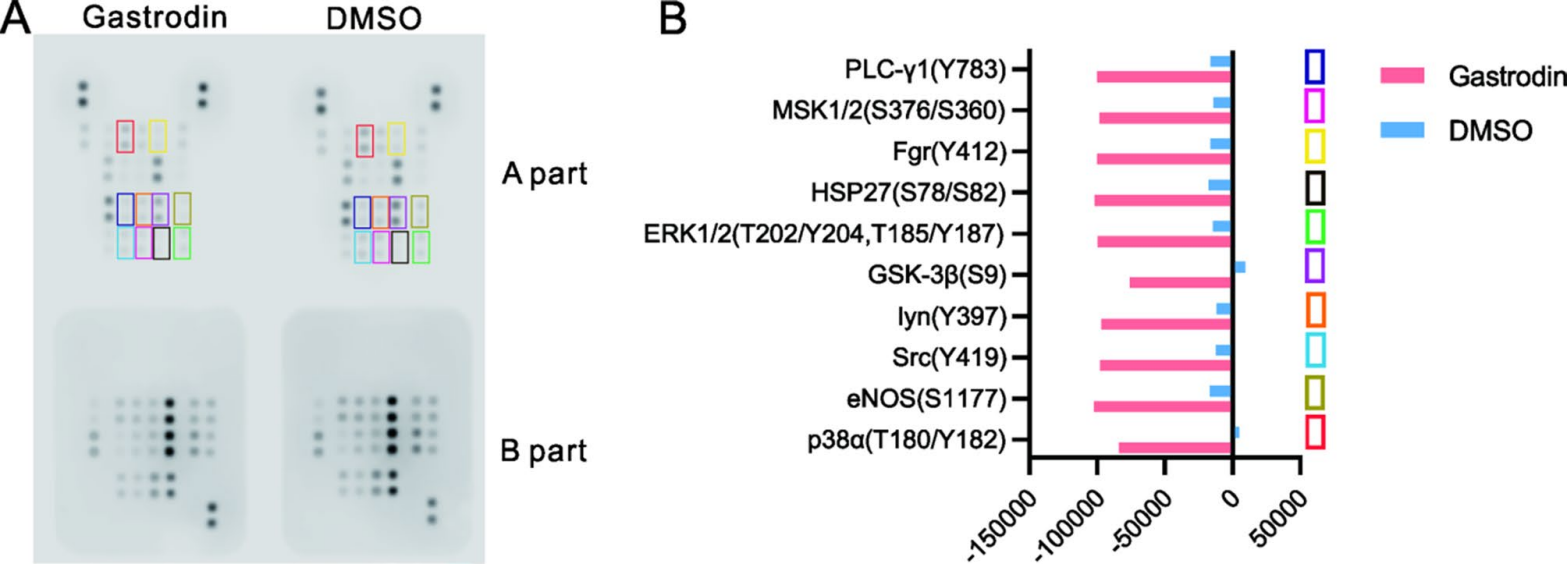
Shows the results of a human phospho-kinase array used to detect phosphorylated proteins in HEK293T cell lysates expressing ataxin-3-77Q. Part **A** of the array was incubated with 200 µg of cell lysate from HEK293T cells expressing ataxin-3-77Q that were either exposed to 50 µM gastrodin or to DMSO. Part **B** shows the top ten proteome profiler “hits” identified in the human phosphokinase array between the gastrodin-treated group and the DMSO-treated group. The data were obtained from two independent experiments

We conducted a protein phosphokinase assay to analyze signaling pathway alterations induced by gastrodin in ataxin-3-77Q-expressing cells. A total of 37 kinases and 2 related total proteins were quantified, with 10 proteins showing differential expression between the gastrodin- and DMSO-treated groups (Table 1; Fig. 4). These differentially expressed proteins were p38α (T180/Y182), eNOS (S1177), Src (Y419), Lyn (Y397), GSK-3β (S9), ERK1/2 (T202/Y204, T185/Y187), HSP27 (S78/S82), Fgr (Y412), MSK1/2 (S376/S360), and PLC-γ1 (Y783). P38 and ERK1/2 serve as the core integrative hubs within this network of ten proteins. Further analysis focused on p38α, ERK1/2, their upstream kinases AKT1/2/3, and their downstream kinase p65, with their expression levels assessed using western blotting.

**Table 1:** The list of proteome profile “hits” identified in the human phosphokinase array in the DMSO-treated and gastrodin-treated groups

Protein name	Phosphorylation site	Mean of differential value	Rank^1^	Mean of relative value to DMSO	Rank^2^
p38α	T180/Y182	88,345	1	3.8	16
eNOS	S1177	85737.25	2	7.4	11
Src	Y419	85212.5	3	23.9	2
lyn	Y397	85,115	4	21.5	3
GSK-3b	S9	84,765	5	-1.8	32
ERK1/2	T202/Y204,T185/Y187	84,740	6	10.0	6
HSP27	S78/S82	84144.25	7	7.2	12
Fgr	Y412	84077.5	8	8.6	9
MSK1/2	S376/S360	83952.75	9	12.1	4
PLC-γ1	Y783	83803.25	10	9.0	7
JNK1/2/3	T183/Y185,T221/Y223	83,295	11	67.6	1
Yes	Y426	83282.5	12	10.1	5
STAT2	Y689	82982.5	13	7.8	10
Lck	Y394	82417.5	14	5.7	14
EGFR	Y1086	82,208	15	5.6	15
PDGF Rβ	Y751	81863.25	16	6.6	13
CREB	S133	80317.5	17	-10.7	34
STAT5a/b	Y694/Y699	76,605	18	-2.6	33
WNK1	T60	74,275	19	-0.2	30
GSK-3a/b	S21/S9	64,150	20	-0.1	29
p53	S46	-46,175	21	-0.8	31
p53	S15	-24,700	22	8.9	8
p53	S392	-17,275	23	-14.4	35
Akt1/2/3	T308	17152.5	24	1.5	20
PRAS40	T246	-14777.5	25	0.5	28
RSK1/2	S221/S227	10382.5	26	1.6	19
PYK2	Y402	6323.75	27	1.8	17
Chk-2	T68	6095	28	1.2	22
STAT3	Y727	-4802.5	29	0.8	27
p70 S6 Kinase	T421/S424	4397.5	30	1.1	24
p70 S6 Kinase	T389	2402.5	31	1.2	23
STAT1	Y701	2392.5	32	1.3	21
RSK1/2/3	S380/S386/S377	1748.75	33	1.1	26
Akt1/2/3	S473	1360	34	1.1	25
c-Jun	S63	1262.5	35	1.7	18

A total of 45 related proteins were included in the assay, but only 32 could be quantified based on the visible signal

The “Mean of differential value” is the average difference between the gastrodin-treated group and the DMSO-treated group. A lower value indicates a decrease in pathway signal

“Rank1” shows the ranking based on the differential values

The “Mean of the relative value to DMSO” is the average relative value of the gastrodin-treated group compared to the DMSO-treated group

“Rank2” shows the ranking based on the relative values to DMSO

“-” indicates no signal in the DMSO-treated group

**Fig. 5:**
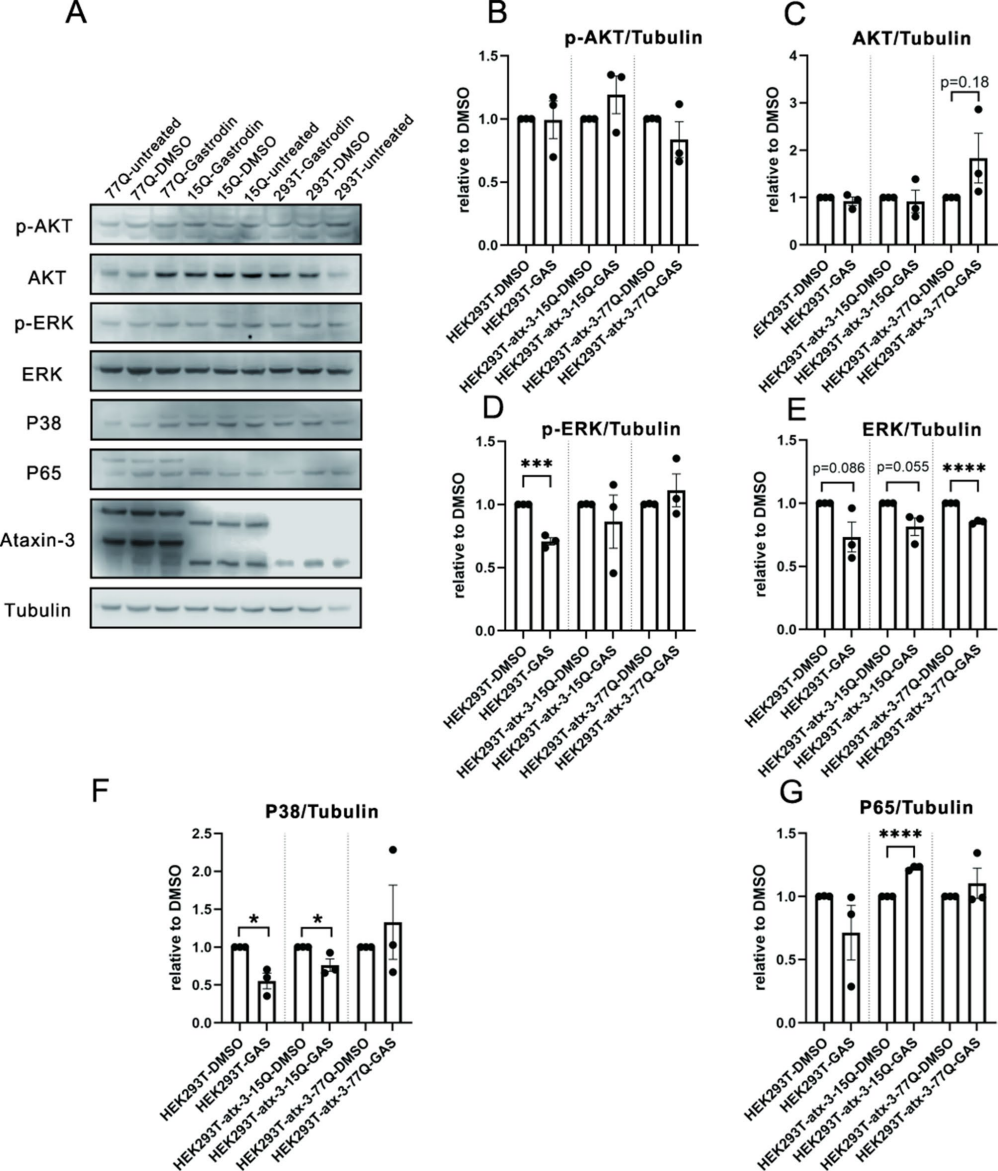
This figure shows differences in protein levels among HEK293T cells, ataxin-3-15Q-expressing HEK293T cells, and ataxin-3-77Q-expressing HEK293T cells. Representative immunoblots and graphs demonstrate protein levels in the AKT, ERK, P38, and P65 pathways. Statistically significant differences are indicated by asterisks (*p < 0.05, ***p < 0.0001, ****p < 0.0001)

The effect of gastrodin on cell models was tested. Gastrodin treatment significantly decreased phosphorylated or total ERK and total p38 protein levels (Fig. 5D-F, Supplementary Fig. 1). Phosphorylated AKT1/2/3 levels did not change with gastrodin treatment (Fig. 5B). Total levels of upstream AKT1/2/3 kinases showed an increasing trend with gastrodin treatment in HEK293T cells expressing ataxin-3-77Q (*P* = 0.18, Fig. 5C). Total levels of p65, a downstream kinase, were significantly increased by gastrodin treatment in HEK293T cells expressing ataxin-3-15Q. However, p65 levels in HEK293T cells expressing ataxin-3-77Q increased after gastrodin treatment without significance (Fig. 5G). These results suggest that the ERK1/2/P38 pathway, especially the total ERK1/2 protein level, was altered by gastrodin treatment in the SCA3 cell model expressing ataxin-3-77Q.

**Fig. 6:**
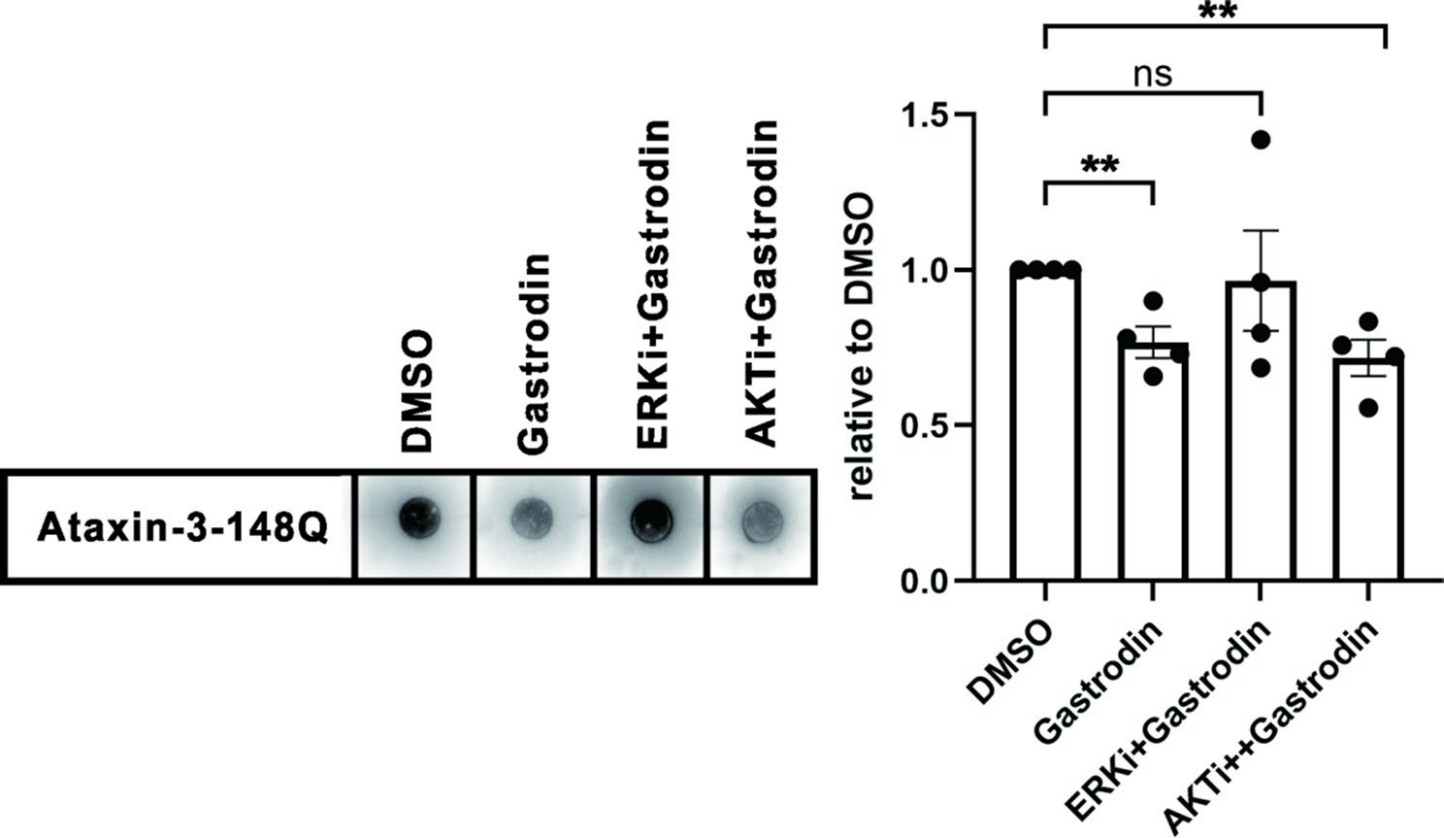
ERK inhibition (Temuterikib, 10nM, CAS: 1951483-29-6) abolishes the suppressive effect of gastrodin on protein aggregation, while AKT inhibition (MK-2206, 1 μm,) does not. (Left) Representative images from the filter trap assay. (Right) Quantitative analysis of protein aggregate levels relative to the DMSO control group (mean ± SEM, *n* = 4 independent experiments). The x-axis indicates different drug treatments; the y-axis shows the relative levels of protein aggregates. Statistical significance was determined by Student’s t-test (**p < 0.01)

To validate whether gastrodin exerts its effects through the ERK pathway, we employed ERK and AKT inhibitors (as controls) in aggregate formation assays. In cell models overexpressing ataxin-3-148Q, gastrodin significantly suppressed protein aggregation compared to the DMSO control. However, inhibition of the ERK pathway abolished the anti-aggregation effect of gastrodin, whereas blocking the AKT pathway did not impair its efficacy (Fig. 6). These results demonstrate that gastrodin-mediated neuroprotection is specifically dependent on the ERK signaling axis.

## Discussion

Gastrodin, the main active ingredient in Gastrodia elata Blume, has been traditionally used to treat dizziness and epilepsy. It also shows potential in protecting against neurodegenerative diseases like Parkinson’s and Alzheimer’s. Previous research has demonstrated that gastrodin can inhibit aggregation and reduce toxicity in diseases like Alzheimer’s and ALS [17]. However, there is currently no information on the relationship between gastrodin and SCA3. This study aimed to investigate the impact of gastrodin on an SCA3 cell model.

### Key mechanistic findings and implications

Gastrodin exhibited no cytotoxicity in SCA3 cell models. Its mechanism of action in treating SCA3 is believed to be related to its antioxidant properties. Gastrodin acts as an antioxidant, helping regulate the formation of aggregates in cells [18]. In our study, 10 µM of gastrodin increased its antioxidant activity in cells. Phosphokinase profiling revealed that gastrodin’s neuroprotective effects in SCA3 cellular models involve modulation of multiple signaling nodes. 10 differentially regulated phosphoproteins were identified in ataxin-3-77Q-expressing cells after gastrodin treatment. Gastrodin may regulate phospho-kinases in the MAPK pathway (ERK1/2, p38α), downstream proteins (MSK1/2, HSP27), Src pathway (Src, Fgr, Lyn), and other pathways (eNOS pathway). The interrelationships between these ten proteins are centered on stress or inflammatory signaling. Src, Lyn, and Fgr act as upstream initiators. They transmit signals through molecules like PLCγ, diverting them into two core pathways: p38 and ERK1/2. These pathways thereby regulate downstream effectors such as MSK1/2, HSP27, GSK3, and eNOS, which collectively orchestrate the cell’s inflammatory, stress, and growth responses. This suggests that gastrodin may be targeting a network hub rather than a single linear pathway, which could explain its potent efficacy. The coordinated downregulation of the ERK1/2-p38 axis is significant, suggesting it as a central pathway influenced by gastrodin. Therefore, gastrodin may play a role in SCA3 by modulating phospho-kinases.

### ERK1/2 suppression as a core mechanism

The MAPK pathway regulates cellular processes such as inflammation, cell stress response, differentiation, and apoptosis. It is implicated in diseases like cancer, immune disorders, and neurodegenerative diseases. Reactive oxygen species can activate proteins in the MAPK pathway [19]. ERK, a member of the MAPK pathway, plays a role in cell proliferation, differentiation, and survival. Studies have shown that gastrodin can reduce pathological ERK overactivation by inhibiting ERK phosphorylation and regulating ERK protein stability. This dual mechanism of action is unique as most kinase modulators target activation states only. Previous research has demonstrated that ERK inhibition can reduce Tau aggregation in Alzheimer’s disease and mitigate TDP-43 toxicity in ALS [20]. Gastrodin has been shown to decrease ERK/JNK MAPK expression and promote Nrf2 expression in cases of hepatotoxicity [21]. Additionally, gastrodin has been found to reduce neurotoxicity in diabetic mice by inhibiting the expression of p-ERK1/2, p-MEK1/2, BDNF, and TrkB [22]. Crucially, pharmacological inhibition of ERK abolished the suppressive effects of gastrodin on protein aggregation. The AKT inhibitor control group did not abolish this effect. These findings suggest that decreased ERK1/2 pathway activation may play a role in the protective effects of gastrodin in SCA3 cell models [23].

### p38 pathway modulation

P38α, a member of the MAPK family, is involved in autophagy and apoptosis. It is activated by dual phosphorylation at Thr180 and Tyr182. Inflamed SCA3 cells show activation of NF-κB, JNK/JUN, and p38/STAT1 pathways, and inhibiting these pathways reduces aggregates in patients with AD or SCA3 [24]. A p38α MAPK inhibitor can clear protein aggregates by acting as a proteasome activator or autophagy inducer in an mTOR-dependent manner [25]. P38α inhibition can also clear aggregates via the Nrf2 pathway [26]. Clinical and preclinical evidence suggests p38α as a potential neurotherapeutic target [27]. Gastrodin may disrupt p38 signaling at the expression level, leading to a reduction in total p38 protein. This suppression could contribute to gastrodin’s cytoprotective effects by potentially inhibiting stress-induced apoptosis in neurodegenerative contexts.

### Upstream/Downstream signaling context

ERK1/2 and eNOS function downstream of AKT [28]. The level of phosphorylated AKT was significantly lower in HEK293T cells overexpressing ataxin-3-15Q compared to control cells, and slightly lower in cells overexpressing ataxin-3-77Q (data not shown). Normal ataxin-3 appeared to increase total AKT protein level, while expanded ataxin-3 decreased it. This suggests that reduced AKT pathway activity may contribute to degeneration in SCA3. The non-significant increase in total AKT without changes in phosphorylation levels suggests AKT may not be the primary target of gastrodin, but could have ancillary roles.

p65, known as RelA, is regulated by p38 [29]. The protein level of p65, a member of the NF-κB family that regulates inflammatory responses, was found to be lower in HEK293T cells overexpressing ataxin-3-77Q compared to wild-type cells (data not shown). Gastrodin increased p65 protein levels in HEK293T cells overexpressing ataxin-3-15Q but not in HEK293T cells or in HEK293T cells overexpressing ataxin-3-77Q. This suggests that polyQ expansion may impair NF-κB activation and warrants further investigation into gastrodin’s role in inflammation regulation across disease states [30]. However, we interpret these observations as potential parallel or ancillary effects of gastrodin treatment rather than as integral components of its primary mechanism.

### Pathway integration and disease relevance

Our findings suggest that the coordinated suppression of ERK1/2 and p38 is in line with the known crosstalk between these MAPK pathways in mediating PolyQ-induced proteotoxicity, transcriptional dysregulation (via p65/NF-κB), and ER stress responses. Gastrodin mitigates proteotoxicity in a SCA3 cellular model by reducing hyperactivated MAPK signaling cascades, particularly ERK/p38 in mutant cells, indicating its potential as a disease-modifying agent.

### Limitations and future perspectives

The study has limitations including the use of HEK293T cells only, and validation in patient-derived neurons is necessary. Validation in animal models of SCA3 is also an essential next step to confirm the therapeutic potential of gastrodin in vivo. Several questions remain unanswered: (1) The exact mechanism of ERK downregulation needs to be clarified, whether it is through proteasomal degradation or transcriptional regulation; (2) Further investigation is needed to understand how polyQ expansion disrupts p65 activation in response to gastrodin; (3) The potential synergistic effects of combining ERK1/2/p38 inhibition with gastrodin treatment need to be systematically evaluated.

## Conclusion

The study suggests that gastrodin can mitigate ataxin-3-77Q toxicity by modulating the ERK1/2/p38/p65 pathway. The decrease in total ERK1/2 protein indicates a new aspect of gastrodin’s pharmacology, showing its potential as a regulator of pathogenic signaling in SCA3. These findings support the potential use of gastrodin in treating SCA3 and offer a new therapeutic approach for patients.

### Methods

#### Cell culture and transfection

Human embryonic kidney 293T (HEK293T) cells were generously gifted by Shaoan Xue from University College London and were maintained as previously described [29]. Three types of plasmids with the pEGFP-N2 vector, pEGFP-N2-MJD15Q, pEGFP-N2-MJD77Q, and pEGFP-N2-MJD148Q were gifted by Thorsten Schmidt from Tuebingen University [30]. Cells were transiently transfected with these plasmids using Lipo8000™ transfection reagent, as previously described [31]. Cells were exposed to gastrodin (CAS: 62499-27-8, Beijing Solarbio Science & Technology Co., Ltd.) or DMSO (CAS: 67-68-5, Beijing Solarbio Science & Technology Co., Ltd.) for different durations after 48 h of transfection.

#### Cell counting Kit-8 (CCK-8) assay and MTT assay

Cells overexpressing ataxin-3-77Q as a SCA3 cell model were used to perform CCK-8 assay (CCK-8; Beyotime, Shanghai, China; Cat No. C0038) as previously described [30]. Cells were seeded into 96-well plates at a density of 20,000 cells per well and exposed to various concentrations of gastrodin (5, 10, 20, 50 and 100µM). DMSO alone were used as the negative control. After treatment, 10 µL of CCK-8 solution was added to each well, followed by incubation at 37 °C for 1 h. The absorbance was measured at 450 nm using an Elx800 microplate reader (Gene Company Limited).

Cell viability was also measured using the MTT assay. Cells were seeded in 96-well plates at 5 × 10³ cells/well and incubated overnight. After treatment with gastrodin (5, 10, 20, 50, and 100 µM) for 4 h, the medium was replaced with 100 µL of fresh medium containing 0.5 mg/mL of MTT and incubated for 4 h at 37 °C. The solution was then carefully removed, and formazan crystals were dissolved in 100 µL of DMSO per well. Absorbance was measured at 490 nm using a microplate reader. Experiments were performed in triplicate.

#### Filter trap assay and aggregate fractionation

A filter trap assay was used to detect SDS-resistant insoluble mutant ataxin-3 aggregates as previously described [31]. Following transfection (72 h), cells were treated with gastrodin for 24 h, then harvested and lysed in PBS containing protease inhibitor (Beyotime, P1005). Lysates were sonicated (1 min, 2 × 30 s cycles). For filter trap assay, samples were mixed with 2% SDS, and 100 µg protein was loaded onto a nitrocellulose membrane (0.45 μm, Merck Millipore) using a filter trap apparatus. After washing twice with PBS, the membrane was subjected to Western blot.

Following the procedure described [16], aggregate fractionation was performed. HEK 293T cells were transiently transfected with normal ataxin-3-15Q and expanded ataxin-3-77Q constructs and treated with gastrodin or DMSO for 24 h. Proteins were then separated and transferred to a nitrocellulose membrane (0.45 μm, Merck Millipore).

#### Proteome profiler human phospho-kinase array

The Proteome Profiler Human Phospho-Kinase Array Kit (ARY003C, R&D Systems) is a membrane-based sandwich immunoassay. The array was divided into two parts (A and B) to maximize sensitivity and minimize cross-reactivity, detecting the relative levels of phosphorylation of 37 kinase phosphorylation sites and 2 related total proteins. At least 1 × 10^7^ HEK293T cells overexpressing ataxin-3-77Q were cultured in the presence or absence of gastrodin for 24 h. The cells were collected on ice and pelleted. Protein phosphorylation was analyzed using this kit according to the manufacturer’s instructions. Signals were measured after adding an enhanced chemiluminescence (ECL) reagent (Affinity, KF8003) using Image Studio™ software on a LICOR instrument (LI-COR Biosciences, Lincoln, Nebraska, USA). All values, minus the average of the reference spot (loading control) values, were divided by two to obtain the final value. Then, the relative values were calculated as the final value in the DMSO-treated group minus the value in the gastrodin-treated group.

#### SDS‒PAGE and western blot

Protein expression was evaluated using western blotting. The process was reported in a previous study [32]. Protein concentrations were determined by a BCA protein assay kit (ZHHC, PQ003). Samples (30 µg) were separated on 10% SDS-PAGE gels and transferred to 0.22 μm nitrocellulose membranes (Merck Millipore). Membranes were blocked with 4% low-fat dry milk in TBST for 2 h, followed by incubation with primary antibody in TBST (2 h at room temperature or overnight at 4 °C). After washing with TBST, blots were incubated with HRP-conjugated secondary antibody for 1 h, washed again, and detected with an Affinity™ ECL kit (KF8003) on a LI-COR instrument (LI‐COR Biosciences, Lincoln, NE, USA).

#### Statistical analysis

All statistical analyses were carried out in GraphPad Prism 9 (La Jolla, CA). The data from multiple independent experiments are expressed as the means ± standard errors (SEMs). Statistical significance was analyzed by Student’s t-test for two groups or one-way ANOVA and multiple comparisons for more than two groups. These data are normally distributed. Significance levels are described as follows: *P* < 0.05*; *P* < 0.01**; *P* < 0.001***, except where noted.

## Electronic Supplementary Material

Below is the link to the electronic supplementary material.

Supplementary Material 1

## Data Availability

We the undersigned declare that this manuscript is original, has not been published before and is not currently being considered for publication elsewhere.
