# Supplementary material for: Gastrodin inhibits the formation of ataxin-3 aggregates by regulating the level of ERK1/2/P38 proteins
Source: Orphanet J Rare Dis. 2026 Feb 19;21:69. doi: 10.1186/s13023-025-04089-1 (PMC12922401; doi:10.1186/s13023-025-04089-1)
Supplement: Supplementary file 1 — Supplementary Material 1 [file 13023_2025_4089_MOESM1_ESM.docx]

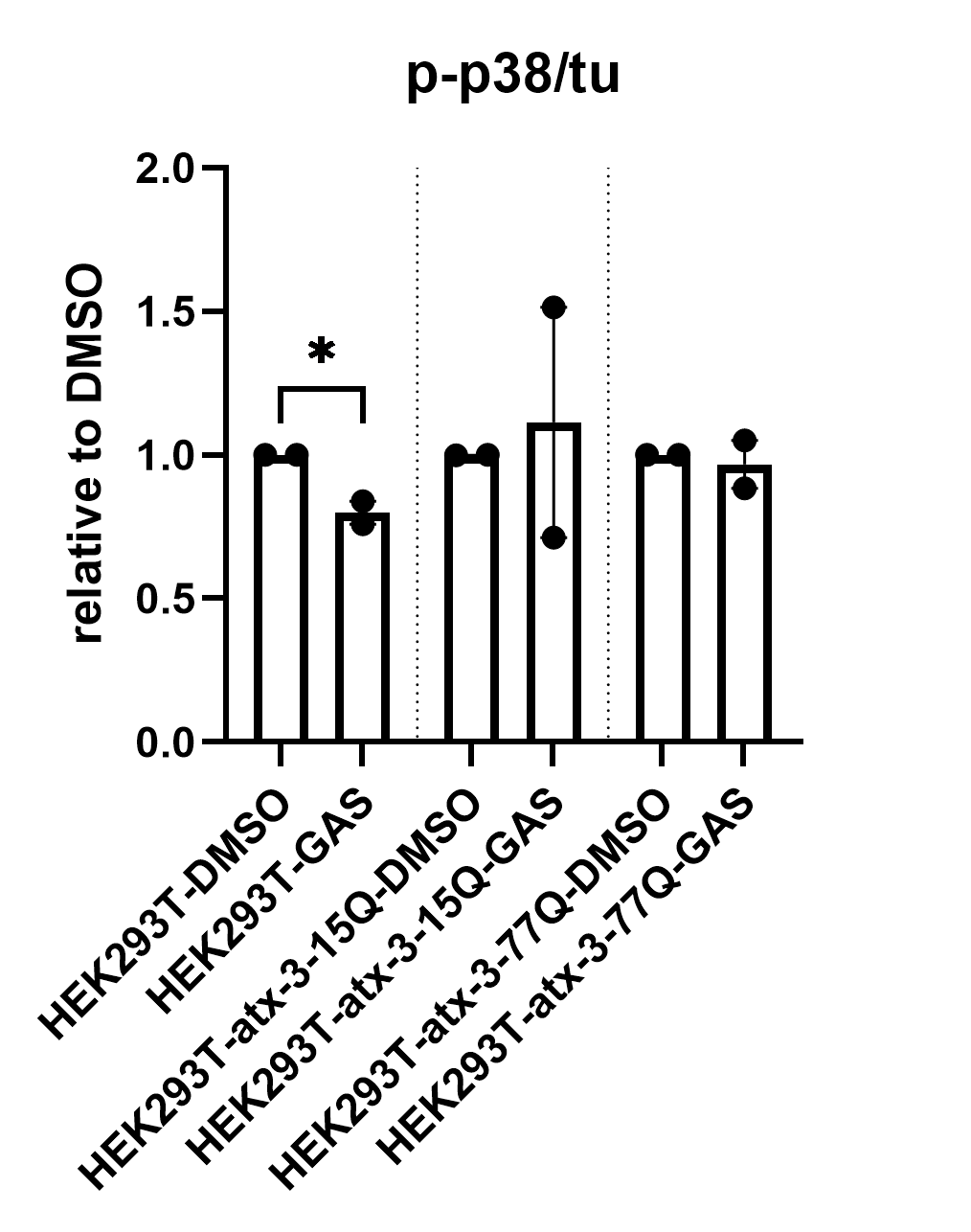


Supplementary Fig. 1 | Phospho-p38 (T180/Y182) levels in HEK293T cell models expressing physiologic (15Q) or pathogenic (77Q) ataxin-3 with or without gastrodin treatment.
